# Alcohol induces α2‐6sialo mucin O‐glycans that kill U937 macrophages mediated by sialic acid‐binding immunoglobulin‐like lectin 7 (Siglec 7)

**DOI:** 10.1002/2211-5463.13919

**Published:** 2024-11-26

**Authors:** Pi‐Wan Cheng, Vishwanath‐Reddy Hothpet, Ganapati Bhat, Kristina Bailey, Lei Li, Derrick R. Samuelson

**Affiliations:** ^1^ Department of Biochemistry and Molecular Biology, College of Medicine University of Nebraska Medical Center Omaha NE USA; ^2^ Fred and Pamela Buffett Cancer Center University of Nebraska Medical Center Omaha NE USA; ^3^ Department of Internal Medicine, College of Medicine University of Nebraska Medical Center Omaha NE USA; ^4^ Department of Chemistry and Center for Diagnostic & Therapeutics Georgia State University Atlanta GA USA; ^5^ Present address: State Forensic Laboratory Bengaluru India; ^6^ Present address: Dayananda Sagar University Bengaluru India

**Keywords:** ethanol, giantin, Siglec 7, U937 macrophages, α2‐6sialo mucin O‐glycans

## Abstract

Alcohol misuse increases infections and cancer fatalities, but mechanisms underlying its toxicity are ill‐defined. We show that alcohol treatment of human tracheobronchial epithelial cells leads to inactivation of giantin‐mediated Golgi targeting of glycosylation enzymes. Loss of core 2 *N*‐acetylglucosaminyltransferase 1, which uses only giantin for Golgi targeting, coupled with shifted targeting of other glycosylation enzymes to Golgi matrix protein 130‐Golgi reassembly stacking protein 65, the site normally used by core 1 enzyme, results in loss of sialyl Lewis x and increase of sialyl Lewis a and α2‐6sialo mucin O‐glycans. The α2‐6sialo mucin O‐glycans induced by alcohol cause death of U937 macrophages mediated by sialic acid‐binding immunoglobulin‐like lectin 7. These results provide a mechanistic insight into the cause of the toxic effects of alcohol and might contribute to the development of therapies to alleviate its toxicity.

## Graphical abstract

Alcohol acts through its metabolite acetaldehyde to inactivate giantin‐mediated Golgi targeting of glycosylation enzymes. This prevents core 2 enzyme from reaching the Golgi complexes and forces other enzymes to target GM130‐GRASP65 site normally used by core 1 enzyme, which leads to formation of α2‐6sialo mucin O‐glycans that kill U937 macrophages. This can help explain the compromised immunity in alcohol abusers.

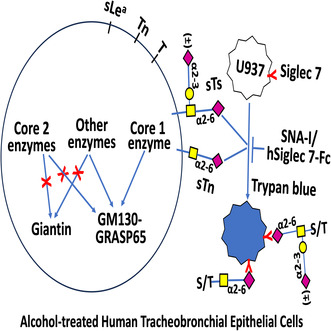

**Abbreviations**
ACH: acetaldehyde
Alda 1: aldehyde dehydrogenase 2 activator 1
ALI culture: air liquid interface culture
ANOVA: analysis of variance
C1GalT1: GalNAc: β3galactosyltransferase 1
C2GnT1: Galβ3GalNAc: (GlcNAc‐GalNAc)β6*N*‐acetylglucosaminyltransferase 1
COSMC: C1GalT1 chaperone protein
DisT: disialyl‐T
ER: endoplasmic reticulum
FuT3‐7: Galβ4GlcNAc:(Fuc‐GlcNAc)α3fucosyltransferases 3–7
GM130‐GRASP65: Golgi matrix protein 130‐Golgi reassembly stacking protein 65
GT: glycosyltransferase
HTBE cells: human tracheobronchial epithelial cells
HTE cells: immortalized human tracheal epithelial cells
Man I: αmannosidase I
Man II: αmannosidase II
Mgat1: *N*‐acetylglucosaminyltransferase 1
Mgat2: *N*‐acetylglucosaminyltransferase 2
OSM: ovine submaxillary mucin
PLA: proximity ligation assay
PNA: peanut agglutinin
ppGalNAcT: peptidyl *N*‐acetylgalactosaminyltransferase
Siglec 7: sialic acid‐binding immunoglobulin‐like lectin 7
sLe^a^
: sialyl Lewis a
sLe^x^
: sialyl Lewis x
SNA‐I: *Sambucus nigra agglutinin‐I*
sT: sialyl‐T
ST3Gal1: Galβ3GalNAcαSer/Thr:α2‐3sialyltransferase 1
ST3Gal3‐6: GlcNAc:α2‐3sialyltransferases 3–6
ST6GalNAc1: GalNAcαSer/Thr:α2‐6 sialyltransferase 1
ST6GalNAc2: Galβ3GalNAcα Ser/Thr:(Neu5Ac‐GalNAc)α2‐6sialyltransferases 2
ST6GalNAc3/4: Neu5Acα2–3Galβ3GalNAcα Ser/Thr:(Neu5Ac‐GalNAc)α2‐6sialyltransferases 3/4
sTn: sialyl‐Tn
TACA: tumor‐associated carbohydrate antigens
Tn: GalNAcαSer/Thr
VVA: *Vicia villosa agglutinin*
β3GnT3: Galβ3GalNAcαSer/Thr: β3*N*‐aceylglucosaminyl‐transferase 3
β4GalT1: GlcNAc: β4galactosyltransferase 1

Alcohol consumption is a worldwide societal phenomenon. Approximately 2 billion people drink alcohol regularly. More than 76 million people suffer from alcohol use disorders, a medical condition characterized by impaired ability to control alcohol use [1]. Alcohol misuse can compromise immune functions [2] and cause severe illnesses in many organs including the heart, liver, pancreas, brain, kidney, bone, colon, and lungs [3, 4]. Alcohol consumption has been linked to infections such as pneumonia and tuberculosis [4] and cancers of the oral cavity, esophagus, larynx, pharynx, female breast, colorectum, stomach, and liver [5, 6, 7, 8]. However, the mechanisms by which alcohol increases the risk for these diseases are ill‐defined.

The toxic effects of alcohol are caused primarily by its carcinogenic metabolite acetaldehyde (ACH) [9]. ACH levels are controlled mainly by the levels of alcohol dehydrogenase, the enzyme which converts alcohol to ACH [10], relative to the levels of aldehyde dehydrogenase, the enzyme that converts ACH to acetic acid [10]. Therefore, the susceptibility of each organ to alcohol toxicity depends largely on the capability of each organ to efficiently reduce ACH [11, 12]. Since alcohol misuse is an addiction, which is very difficult to overcome, there is a pressing need to develop approaches to alleviate alcohol‐associated toxicity. To accomplish that, a better understanding of the mechanisms of the toxic effects of alcohol is needed.

Alcohol has been shown to increase sialylated mucins in rat gut mucosa [13, 14]. However, the specific sialo mucin O‐glycans have never been identified. Because certain sialo mucin O‐glycans can compromise immune functions [15, 16] by serving as the ligands of sialic acid‐binding immunoglobulin‐like lectin 7 (Siglec 7) [17], a member of the inhibitory Siglec family [18, 19], identification of these sialo mucin O‐glycans induced by alcohol could help advance our understanding of the link of these mucin O‐glycans to alcohol‐associated toxicity. Recently, we have found that as cancer advances to malignant stage, loss of giantin functions as a major Golgi matrix protein involved in the maintenance of Golgi morphology [20] and the primary Golgi targeting site for endoplasmic reticulum (ER)‐derived vesicles [21, 22, 23, 24, 25] results in fragmentation of the Golgi complexes [22, 25] as well as alteration of both N‐glycans [23, 24, 26, 27, 28] and mucin O‐glycans [22, 29, 30, 31, 32, 33, 34]. Because alcohol exposure also causes Golgi fragmentation [35] and altered N‐glycosylation [36], we decided to examine if alcohol exposure also induces formation of the sialo mucin O‐glycans that can compromise immune functions.

## Materials and methods

### Materials

The materials used in this study and their suppliers are listed below. Unless specificied, United States is where the suppliers located. Absolute ethanol, (Cat no. 64‐17‐5) Decon laboratories, King of Prussia, PA; Aldehyde dehydrogenase 2 activator‐1 (Alda‐1), provided by Dr. Dalia Mochly‐Rosen at Stanford University, Stanford, CA; (±)blebbistatin, Sigma (Cat. no. 1760), St. Louis, MO; BronchiaLife™ Epithelial Airway Medium Complete Kit, Lifeline Cell Technology, Walkersville, MD (Cat. no, LS‐1047 & LL‐0023): Rabbit pAb‐giantin (Cat no. ab24586), rabbit pAb‐ST3Gal1 (Cat no. ab96129), mouse mAb‐Man IA (Cat no. ab140613), rabbit mAb‐GM130 (Cat no. ab52649), mouse pAb‐GM130 (Cat no. ab169276), and rabbit mAb‐GRASP65 (Cat no. ab174834), Abcam, Cambridge, UK; Rabbit pAb‐β3GnT3 (Cat no. GTX108928), GeneTex, Hsinchu, Taiwan; Mouse pAb‐C2GnT1 (Cat no. H00002650‐B01P), Abnova, Taipei, Taiwan; Mouse, mAb‐sLe^a^ (Cat no. MAB2095), Millipore, Billerica, MA; Mouse mAb‐GRASP65 (Cat no. sc365434) and mouse mAb‐CD15s (sLe^x^) (Cat no. sc32243), Santa Cruz Biotechnology, Dallas, TX; Donkey Alexa Flour‐488‐conjugated pAbs mouse IgG and IgM (Green) (Cat no. 715‐546‐150 and Cat no. 115‐485‐075) and donkey DyLight‐594‐conjugated pAbs‐ Ab‐rabbit IgGs (Cat no. 711‐585‐152), Jackson‐Immuno‐Research Laboratories, West Grove, PA; SNA‐I (Cat no. L1300), FITC‐labeled SNA‐I (Cat no. NC1365364), VVA (Cat no. F 46012), PNA (Cat no. FL 1071) and VVA (Cat no. F 4601) lectins, EY laboratories, San Mateo, CA; Acetaldehyde (Cat no. AA33244AE) and 4‐methylpyrazole (Cat no. AC206970010), rabbit Abs‐mouse IgG (Cat no. 31188), Fisher Scientific, Pittsburgh, PA; Nonspecific neuraminidase (Cat no. P0720), New England Biolabs, Ipswich, MA; Bronchial epithelial growth medium, (Cat no. SC3211b) Lonza, Basel, Switzerland; PneumaCult ALI media, (Cat no. 00193514) StemCell Technologies, Inc., Cambridge, MA; DAPI, (Cat no. DU092202) Invitrogen, Waltham, MA; Duolink PLA kit, (Cat no. 05005) Sigma, St. Louis, MO; Collagen‐coated 6.5‐mm transwell inserts (0.4‐μm pore) (Cat no. 3495), Corning, Kennebunk, ME; U937 macrophages (RRID: cvcl_007) by Joyce Solheim at the University of Nebraska Medical Center, Omaha, NE, USA; Immortalized human tracheal epithelial (HTE) cells [37, 38] by Dr. Reen Wu at the University of California at Davis, CA, USA; PMA, phorbol 12‐myristate 13‐acetate, Fisher Scientific. Ovine submaxillary mucin was isolated as described [39]; sialyl‐Tn‐Ser and disialyl‐T‐Ser were synthesized as described [40].

### ALI cultures of HTBE cells and cultures of immortalized HTE cells

Normal HTBE cells were isolated from deidentified human lungs that were not used for transplantation. We accepted lungs from the International Institute for the Advancement of Medicine and the Live on Nebraska, the local organ retrieval service. We excluded donors with a history of any lung disease, current smoking, ≥ 20 pack‐year history of smoking, and heavy alcohol use (> 2 standard drinks a day for women and > 3 for men). The protocol was approved by the International Institute for the Advancement of Medicine and Live on Nebraska ethics committees and was deemed nonhuman subjects research by the University of Nebraska Medical Center Institutional Review Board (IRB#318‐09‐NH). HTBE cells were isolated using a method previously described [41, 42]. Briefly, the tracheal and bronchial airways were dissected out and protease digested for 36–48 h. Then, the airway lumens were scraped, and the resulting cells were plated on collagen‐coated plates in bronchial epithelial growth medium. They were subsequently transferred to collagen‐coated 6.5‐mm transwell inserts (0.4‐μm pore) at ALI using PneumaCult ALI media (StemCell). After the cells became confluent, they were transitioned to ALI and cultured until they were fully ciliated. Cells from a minimum of five different donors were used in each experiment. Immortalized HTE cells were cultured on T‐25 flasks in BronchiaLife™ epithelial airway medium.

### Treatment of ALI cultures of HTBE cells with EtOH, 4‐methylpyrazole, Alda‐1, and blebbistatin

The ALI cultures of HTBE cells were treated for 72 h with ethanol with or without other agents by bathing the inserts in PneumaCult ALI media containing ethanol at concentrations as indicated in each experiment in the absence or presence of 5 mm 4‐methylpyrazole, 50 μm Alda‐1, or 35 μm blebbistatin. The conditioned media were replaced every 24 h.

### Confocal immunofluorescence microscopic analysis of giantin, C2GnT1, ST3Gal1, and β3GnT3 in paraffin sections of ALI cultures of HTBE cells

At the end of the treatment protocols as described above, ALI cultures of HTBE cells were fixed in 4% paraformaldehyde and then embedded in paraffin. Paraffin sections at 5 μm thickness were deparaffinized in 100% xylene for 10 min and hydrated in sequential series of ethanol solution (100%, 95%, 70%, and 50%) for 10 min each. Then, the slides were incubated in 1× PBS for 20 min at 37 °C followed by washing twice with 1× PBS for 5 min each. To perform immunostaining of giantin and C2GnT1, the slides were incubated with rabbit antibodies against giantin (1 : 100) and mouse antibodies against C2GnT1 (1 : 100) at 37 °C for 1 h followed by treatment with DyLight‐594‐conjugated donkey Abs‐rabbit IgG and IgM (1 : 200) and Alexa Fluor‐488‐conjugated donkey Ab‐mouse IgG (1 : 200) for 1 h at RT and then mounted in ProLong Gold antifade reagent with DAPI (Invitrogen). To perform immunostain of ST3Gal1 and β3GnT3, the paraffin sections were treated with rabbit antibodies again ST3Gal1 (1 : 100) or β3GnT3 (1 : 100) followed by DyLight‐594‐conjugated donkey Abs‐rabbit IgG (1 : 200). Slides processed without primary‐Abs served as negative control. The cells were imaged by confocal fluorescence microscopy (Jena, Germany) using laser excitation wavelengths of 405 nm (blue), 488 nm (green), 594 nm (red) and emission wavelengths of 421 nm (blue), 519 nm (green), and 618 nm (red), respectively. The images of each slide were analyzed by imagej at 5 random areas under a Zeiss 710 microscope.

### FITC‐VVA and PNA lectin stain of paraffin sections of ALI cultures of HTBE cells

Paraffin sections of ALI cultures of HTBE cells prepared as described above were processed for lectin stain. Control slides were covered for 1 h at 37 °C with 200 μL GlycoBuffer while slides to be treated with neuraminidase were covered with same buffer containing 200 units of neuraminidase. Then, the slides were washed twice with PBS followed by incubation with 1% BSA in PBS for 1 h before exposure to FITC‐VVA or PNA lectins (20 μg·mL^−1^ in serum‐free medium) at 37 °C for 1 h and then mounted in ProLong Gold antifade reagent with DAPI (Invitrogen). The green fluorescence images were captured by imagej under a Zeiss 710 microscope.

### *In situ* proximity ligation assay (PLA) of colocalization of Man IA with giantin versus GM130/GRASP65

Duolink PLA kit (Sigma) was employed to perform *in situ* PLA to determine close proximity between two target molecules [23, 24]. Briefly, HTBE cells in deparaffinized tissue sections were washed thrice with PBS and fixed in 4% formaldehyde followed by blocking using 1% donkey serum. Then, the cells were incubated with two primary antibodies prepared from different animal species, which recognize two different target molecules of interest. Subsequently, the cells were incubated with the secondary antibodies conjugated with two respective complementary oligos (Plus and Minus), followed by ligation and amplification. Red fluorescent spots (PLA‐signal) that indicate a close proximity between these two target molecules were examined by confocal fluorescence microscopy. The signal obtained by primary‐Abs from different species against two different motifs of same target was used as the positive control and the sample prepared with only one primary Ab was the negative control. To evaluate the colocalization of Man IA with giantin, GM130, or GRASP65, PLA was performed using mouse anti‐Man IA antibodies plus rabbit anti‐mouse secondary antibody conjugated with a Plus oligo nucleotide probe, and rabbit pAb‐Giantin, rabbit mAb‐GM130, or rabbit mAb‐GRASP65, conjugated with a Minus oligo nucleotide probe prepared using Duolink *in situ* probe maker kit (Sigma). BSA (1%) was used to block background signal.

### Live cell lectin stain of immortalized HTE cells

Immortalized HTE cells plated on culture inserts which were treated with 50 mm EtOH for 30–36 h or untreated control cells were exposed to FITC‐SNA‐I (20 μg·mL^−1^) in 1% BSA. After incubation at RT for 2 h, the cells were rinsed with PBS 3× and then fixed in buffered 5% formaldehyde for 10 min before mounted with antifade DAPI and examined under a confocal microscope.

### Viability assay of U937 macrophages

U937 macrophages were cultured in RPMI medium containing 10% FBS and 100 units/ml of Penicillin and streptomycin and exposed to phorbol 12‐myristate 13‐acetate (PMA) overnight prior to the viability assay. To perform the viability assay of U937 cells exposed to EtOH‐treated HTE cells, U937 cells (5 × 10^5^ mL^−1^) were cocultured at 37 °C and 5% CO_2_ with HTE cells, which had been cultured in a 48‐well plate in medium with or without 50 mm EtOH for 30–36 h and then with or without 10 μg·mL^−1^ SNA‐I in EtOH‐free media for 1 h. After cocultured for 3–4 h, U937 cells were harvested and analyzed for viability by trypan blue exclusion. To perform the viability assay of U937 cells exposed to α2‐6sialo mucin O‐glycans, the PMA‐treated U937 cells (5 × 10^5^ mL^−1^) were exposed to (a) OSM (30 μg·mL^−1^) with or without SNA‐I (10 μg·mL^−1^) pretreatment at 37 °C and 5% CO_2_ for 1 h (*n* = 3) or (b) OSM (30 μg·mL^−1^), sTn‐Ser (10 μg·mL^−1^), or DisT (10 μg·mL^−1^) with or without rhSiglec Fc (10 μg·mL^−1^) pretreatment at 37 °C and 5% CO_2_ for 1 h (*n* = 3). After incubation at 37 °C and 5% CO_2_ for 3–4 h, the U937 cells were harvested and measured for viability based on trypan blue exclusion.

### Image quantification and data analysis

The figures were aligned in Microsoft power point presentation. The fluorescence images in each slide were analyzed at five random areas using NIH imagej. The data are expressed as mean ± SD. Statistical analysis of the significance of the difference between the means of the groups was performed by ANOVA.

## Results

### Alcohol effects on Golgi morphology and intracellular localization and levels of core 2 *N*‐acetylglucosaminyltransferase 1 (C2GnT1) in human tracheobronchial epithelial (HTBE) cells

**Fig. 1:**
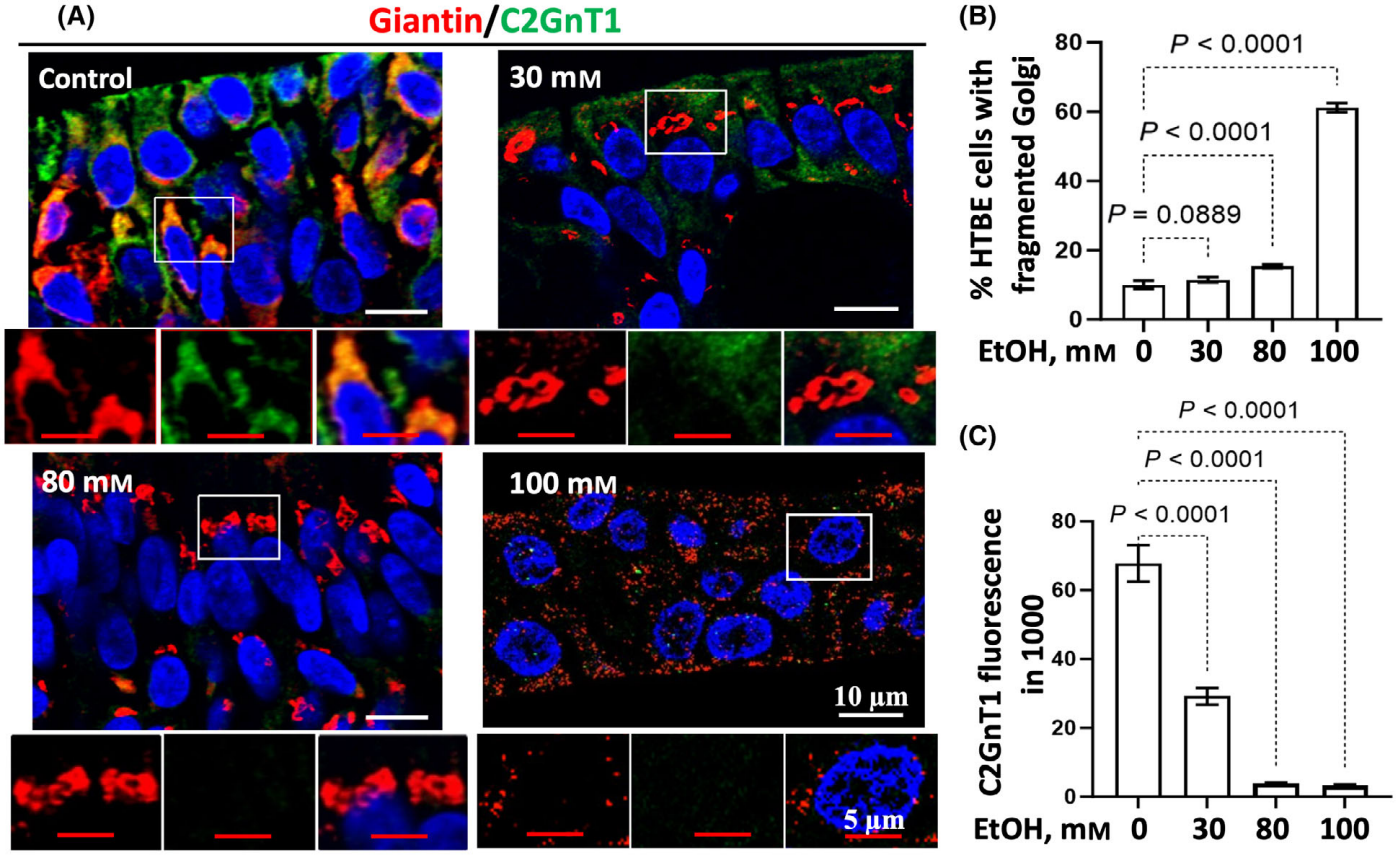
Ethanol concentration effects on Golgi morphology, and intracellular distribution and levels of C2GnT1 in HTBE cells. (A) Confocal immunofluorescence images of Golgi (giantin‐red) and C2GnT1 (green) in the paraffin sections of ALI cultures of HTBE cells exposed to 30–100 mm ethanol for 72 h, (B) % cells with fragmented Golgi, and (C) intracellular levels of C2GnT1 measured by imagej program at five randomly chosen regions and expressed as mean ± SD. Statistical analysis was performed by ANOVA.

Golgi morphology and intracellular localization and levels of C2GnT1 [43] in air liquid interface (ALI) cultures of HTBE cells treated with 0, 30, 80, or 100 mm EtOH for 72 h were assessed by confocal immunofluorescence microscopy following immunostaining of C2GnT1 (green) and giantin (red). The Golgi morphology was not affected at 30 mm EtOH but was significantly fragmented at 80 mm EtOH and further fragmented at 100 mm EtOH (Fig. 1A,B). C2GnT1 was already excluded from the Golgi and reduced in staining intensity in cells treated with 30 mm EtOH. The intracellular C2GnT1 protein was greatly depleted in cells treated with 80 and 100 mm EtOH (Fig. 1A,C).

### Alcohol effects mediated by its metabolite ACH and prevented by co‐treatment with 4‐methylpyrazole, acetaldehyde dehydrogenase 2 activator 1 (Alda‐1), or blebbistatin

**Fig. 2:**
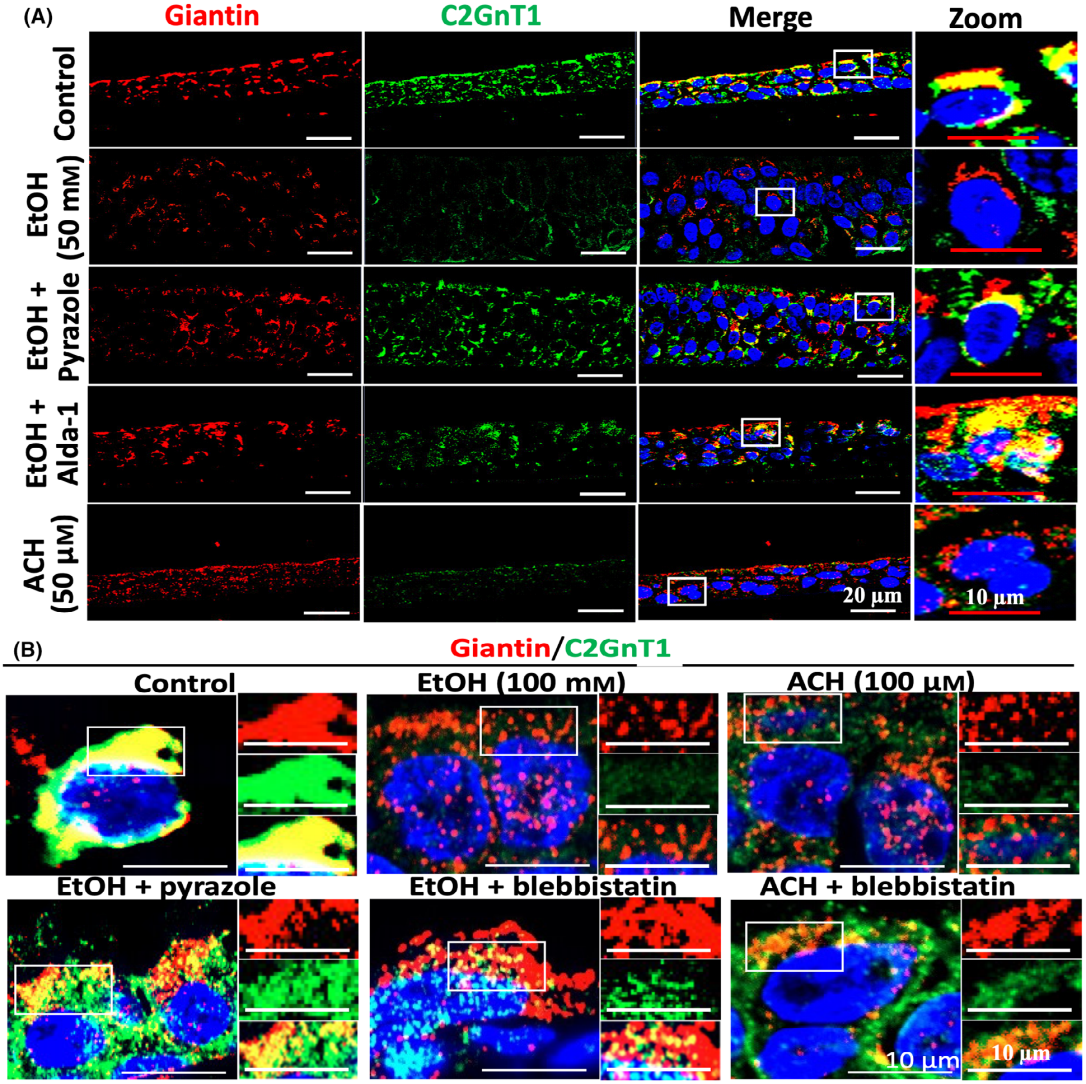
Confocal immunofluorescence images of ALI cultures of HTBE cells treated with alcohol or ACH with or without inhibitors or activator. Paraffin sections of HTBE cells were treated with anti‐giantin Ab (red), anti‐C2GnT1 Ab (green), and DAPI after the cultured cells had been treated with (A) 50 mm ethanol in the absence or presence of 5 mm 4‐methylpyrazole or 50 μm Alda‐1, or with 50 μm ACH, and (B) 100 mm ethanol ± 5 mm 4‐methylpyrazole or 35 μm blebbistatin, or 100 μm ACH ± 35 μm blebbistatin.

The alcohol‐associated toxicity on cells and tissues is driven primarily by its primary metabolite ACH [9, 10]. We examined if ACH reproduced the effects of alcohol and reduction of ACH prevented the effects of alcohol. As shown in Fig. 2A, treatment of HTBE cells with 50 μm ACH reproduced the alcohol effects, that is, outside Golgi distribution and reduced intracellular content of C2GnT1. Treatment of 50 mm EtOH‐exposed HTBE cells with alcohol dehydrogenase inhibitor 4‐methylpyrazole [44] or Alda‐1 [45] mitigated the alcohol effects. Further, 4‐methylpyrazole treatment of HTBE exposed to 100 mm EtOH also preserved the intact Golgi morphology as well as Golgi localization and intracellular level of C2GnT1 (Fig. 2B). Finally, the toxic effects of EtOH or its metabolite were also prevented by co‐treatment with blebbistatin, an inhibitor of non‐muscle myosin IIA involved in Golgi fragmentation in malignant cancer cells [22].

### Alcohol effects on giantin‐mediated Golgi targeting of glycosylation enzymes

**Fig. 3:**
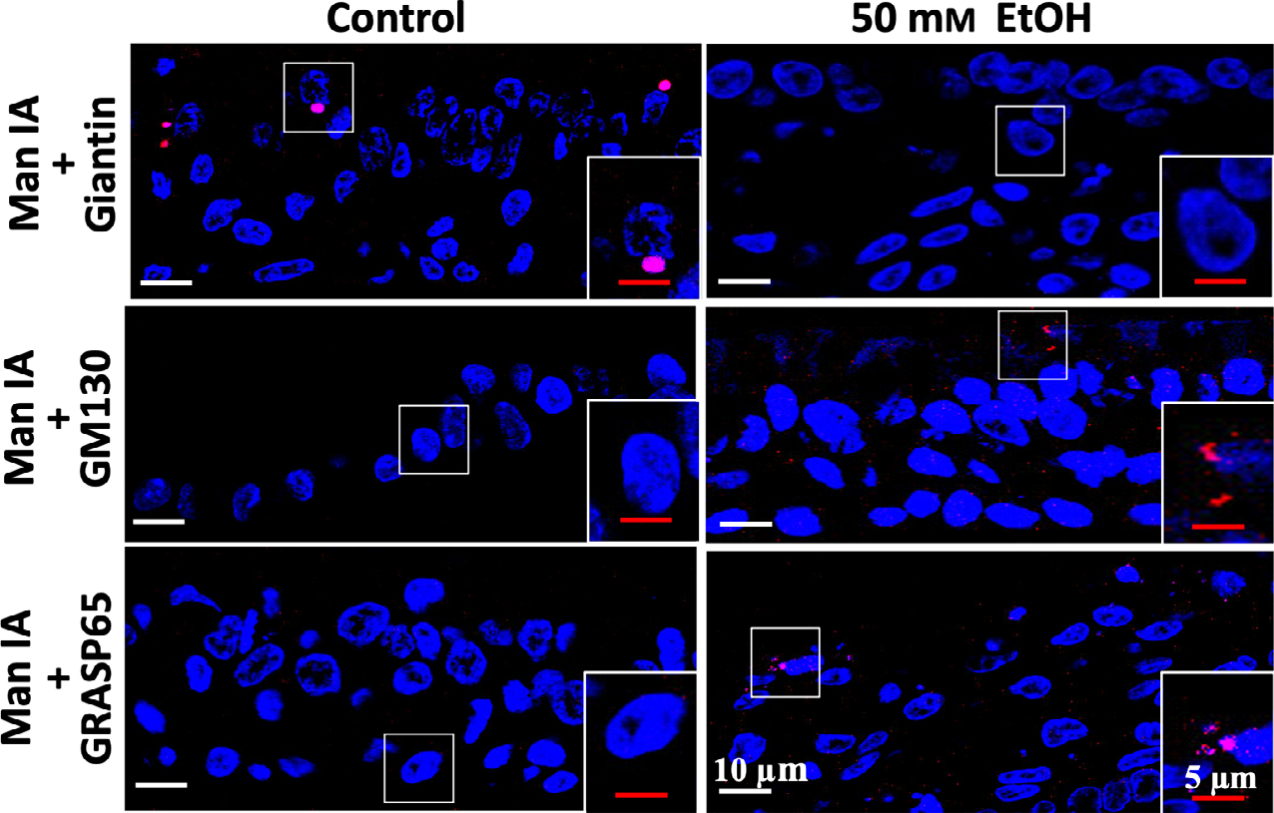
Confocal immunofluorescence images of *in situ* Proximity Ligation Assay of Man IA colocalization with giantin vs. GM130 and GRASP65 in HTBE cells with or without ethanol treatment. The analysis was performed on paraffin sections of ALI cultures of HTBE cells exposed to 0 or 50 mm ethanol.

To assess whether loss of C2GnT1 was the result of its failure to target the giantin site and whether this effect also occurred to other glycosylation enzymes which also use giantin for targeting, we determined the Golgi localization of Man IA at giantin versus Golgi matrix protein 130‐Golgi reassembly stacking protein 65 (GM130‐GRASP65) by *in situ* Proximity Ligation Assay (PLA) as we previously described [23, 24]. We found that EtOH treatment shifted Golgi localization of Man IA from giantin to GM130‐GRASP65 (Fig. 3), confirming that alcohol exposure inactivated giantin‐mediated Golgi targeting, which led to loss of C2GnT1 (Figs 1 and 2) because C2GnT1 only uses giantin for Golgi targeting [21, 22] while Man IA and other glycosylation enzymes still reached Golgi using GM130‐GRASP65 site for targeting [23, 24].

### Alcohol effects on mucin O‐glycans produced in HTBE cells

**Fig. 4:**
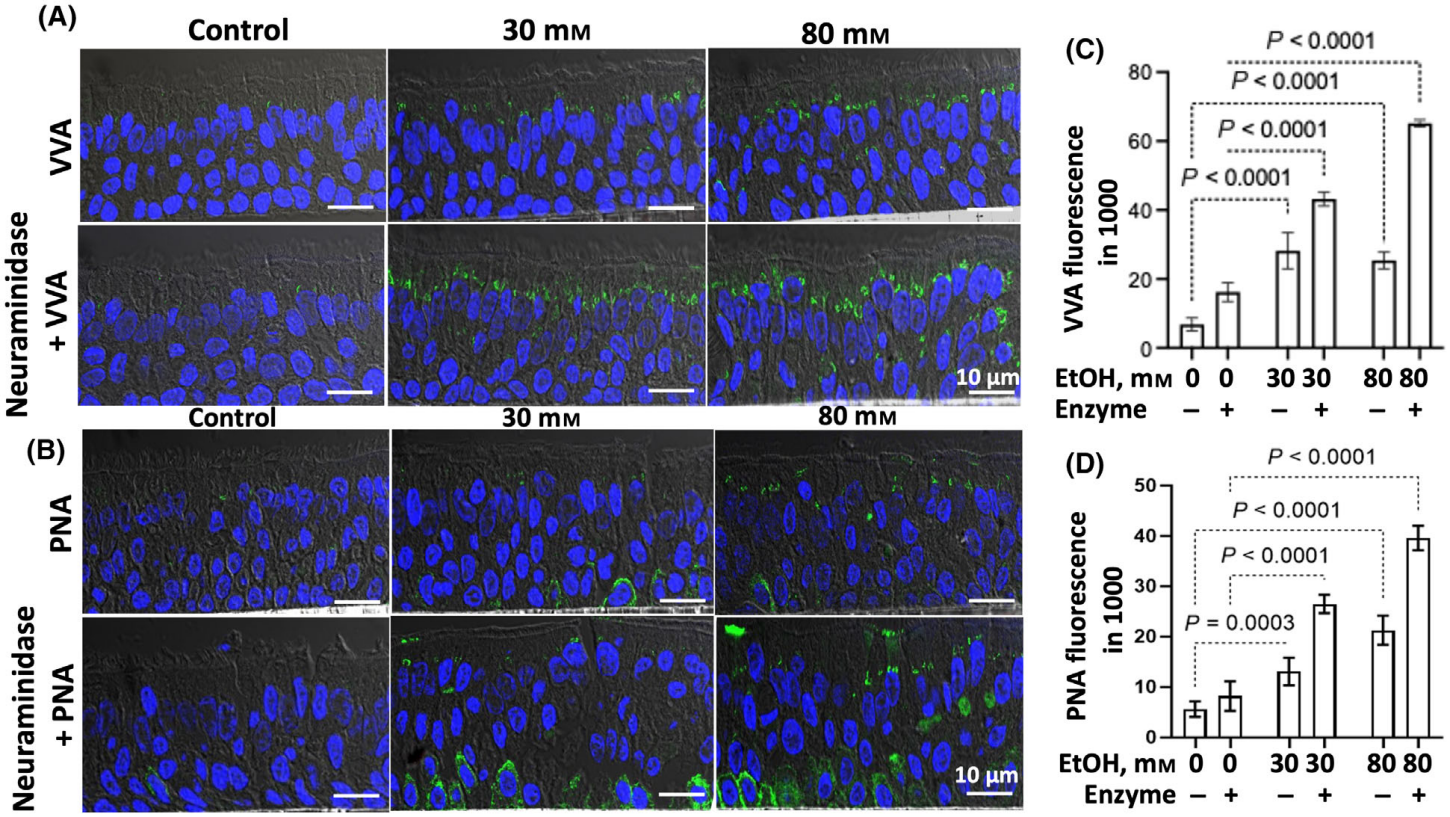
VVA and PNA lectin stain of HTBE cells treated with EtOH. Confocal lectin fluorescence images of (A) FITC‐PNA and (B) FITC‐PNA ± neuraminidase‐treated paraffin sections of HTBE cells that had been exposed to 0, 30, or 80 mm EtOH. The fluorescence intensity in (A) and (B) was measured at five randomly chosen areas and expressed as mean ± SD shown in (C) and (D), respectively. The statistical analysis was performed by ANOVA.

**Fig. 5:**
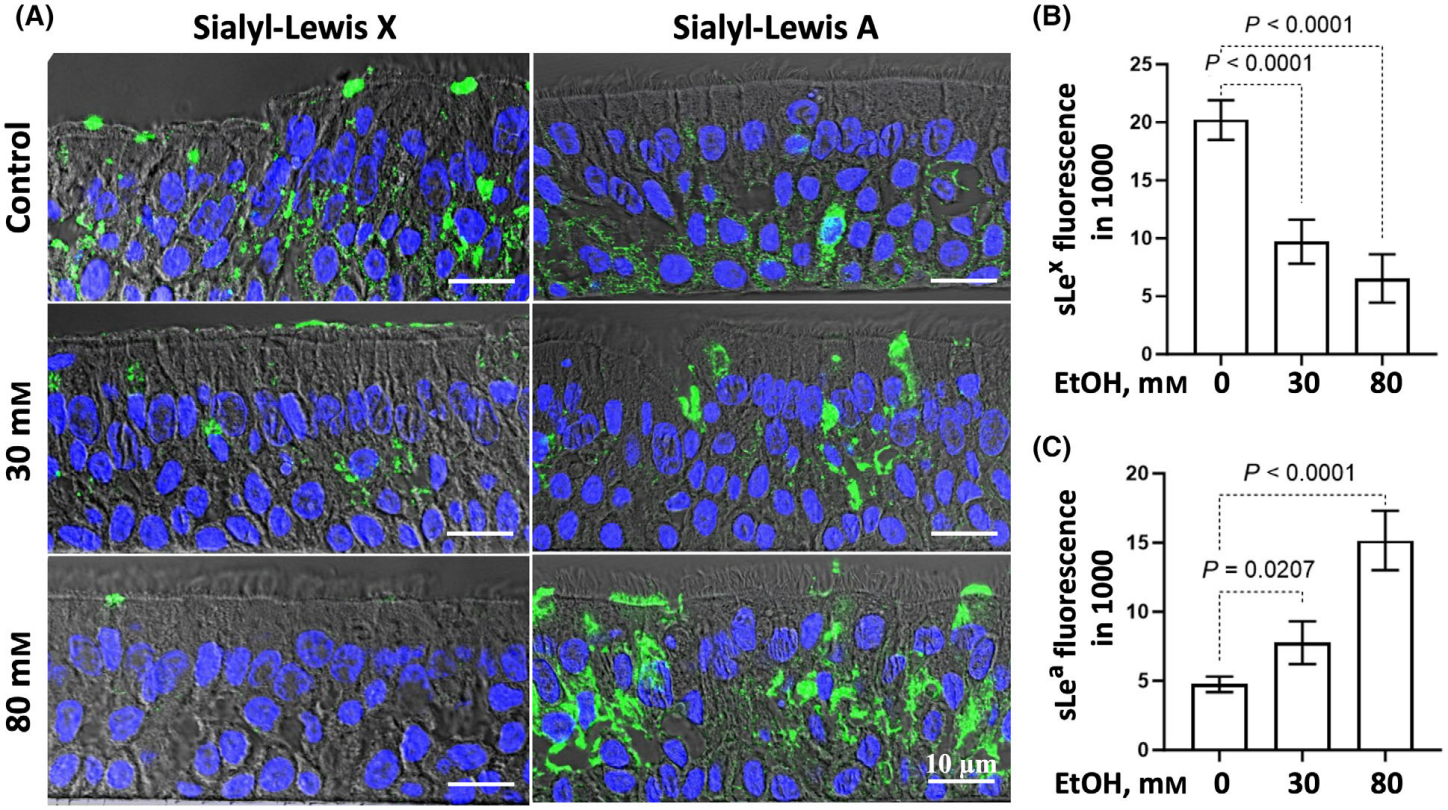
Immunostaining of sLe^x^ and sLe^a^ in HTBE cells treated with EtOH. (A) Confocal immunofluorescence images of sLe^x^ and sLe^a^ in paraffin sections of ALI cultures HTBE cells treated with anti‐sLe^x^ and sLe^a^ antibodies and DAPI after the cultured cells had been exposed to 0, 30, or 80 mm EtOH for 72 h. The immunofluorescence intensities of (B) sLe^x^ and (C) sLe^a^ were measured at five randomly chosen areas by imagej and expressed as mean ± SD. The statistical analysis was performed by ANOVA.

Next, we examined the effects of alcohol‐induced loss of giantin‐mediated Golgi targeting and C2GnT1 on several mucin O‐glycans. Tn and sialyl‐Tn (sTn) on EtOH‐treated HTBE cells were assessed by FITC‐*Vicia villosa agglutinin* (VVA) [46] stain before and after neuraminidase treatment. Also, T antigen and sialyl‐Ts (sTs) were assessed by FITC‐*peanut agglutinin* (PNA) [46] stain before and after neuraminidase treatment. The difference between with and without neuraminidase treatment represents sTn and sTs, which include 3'sialyl‐T (3'sT), 6sialyl‐T (6sT), and disialyl‐T (DisT), respectively. As shown in Fig. 4, treatment of HTBE cells with 30 or 80 mm EtOH increases Tn, sTn, T, and sTs, which are summarized in Table S1. EtOH treatment of HTBE cells also resulted in loss of sialyl Lewis x (sLe^x^) and increase of sialyl Lewis a (sLe^a^) (Fig. 5).

### Effect of alcohol‐treated HTE cells on the viability of U937 macrophages

**Fig. 6:**
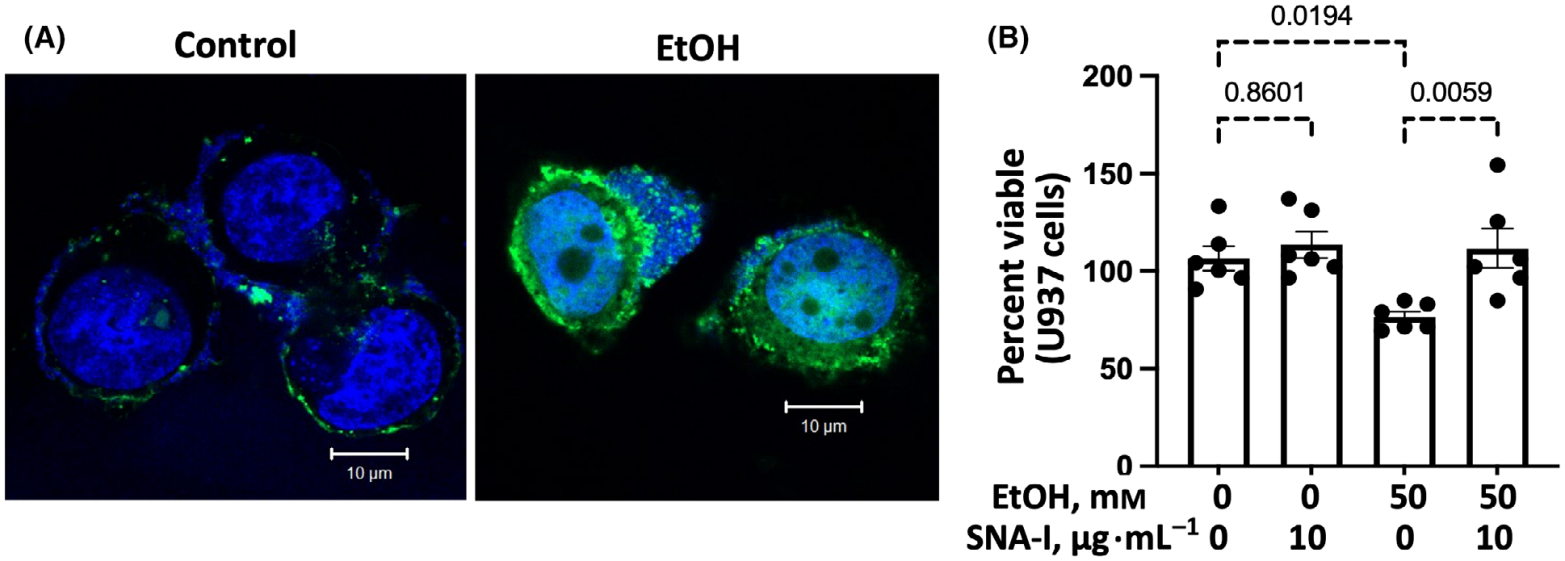
Confocal SNA‐I lectin fluorescence images of 50 mm alcohol‐treated HTE cells stained with (A) FITC‐SNA‐I and measured for fluorescence intensity at five random areas by imagej and expressed as (B) mean ± SD. The statistical analysis was performed by ANOVA.

U937 macrophages were killed after exposure to alcohol‐treated HTE cells (Fig. 6), which was prevented by pretreatment of the cells with α2‐6sialic acid‐specific *Sambucus nigra agglutinin*‐I (SNA‐I).

### Effects of ovine submaxillary mucin (OSM), sTn, or DisT on the viability of U937 macrophages

**Fig. 7:**
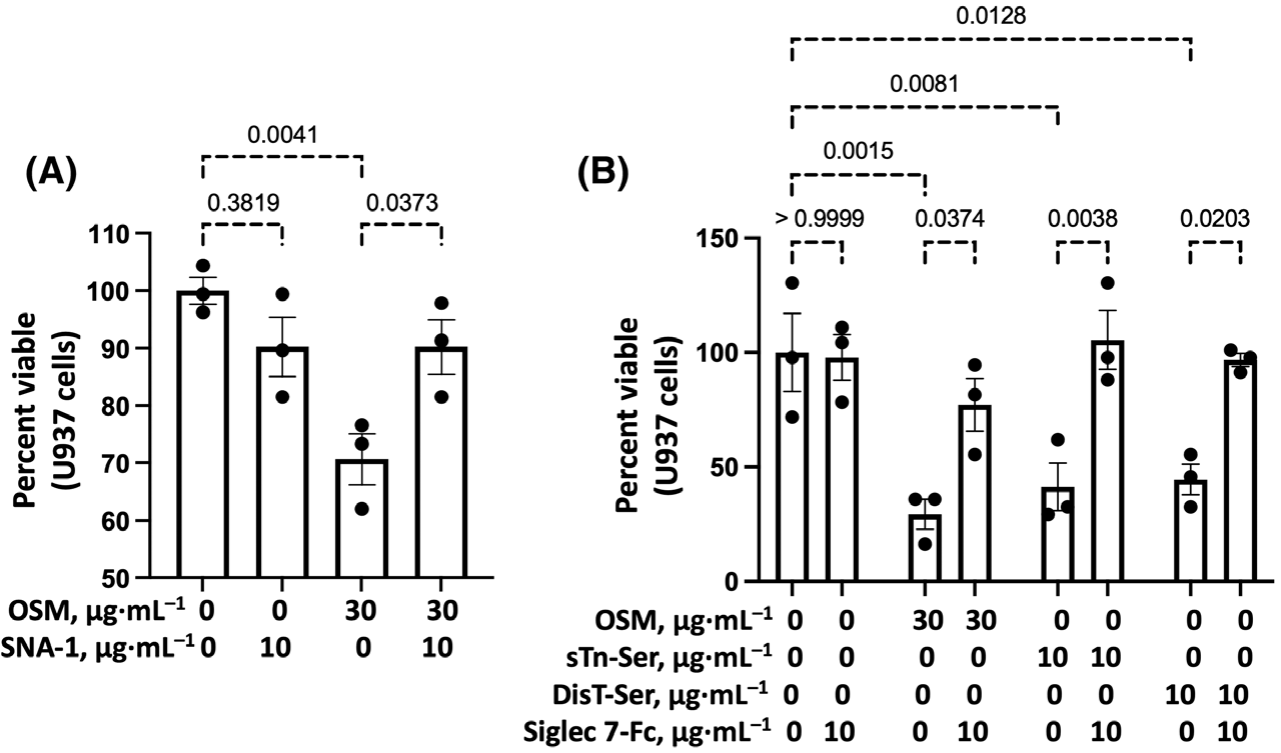
(A) SNA‐I inhibition of the killing of U937 macrophages by OSM and (B) Siglec 7‐Fc inhibition of the killing of U937 macrophages by OSM, sTn‐ser, or DisT‐ser. The viability of U937 cells was measured by trypan blue exclusion after exposure for 3–4 h to (A) culture medium or OSM with or without SNA‐I or (B) culture medium, OSM, sTn‐ser, or DisT‐ser with or without Siglec 7‐Fc. Statistical analysis (*n* = 3) was performed by ANOVA.

U937 macrophages were killed after exposure to sTn‐rich OSM [39] (Fig. 7A). The OSM‐mediated killing of U937 cells was prevented by pretreatment of OSM with SNA‐I. U937 macrophages were also killed after exposure to sTn‐Ser or DisT (Fig. 7B). The killings of U937 macrophages by these α2‐6sialo mucin O‐glycans were prevented by pretreatment of these glycans with Siglec 7‐Fc.

## Discussion

In this study, we have found that alcohol acts through its metabolite ACH to inactivate giantin‐mediated Golgi targeting of glycosylation enzymes in HTBE cells, which leads to altered glycosylation and Golgi fragmentation. We have shown that the α2‐6sialo mucin O‐glycans induced by alcohol kill U937 macrophages in a Siglec 7‐dependent manner. Together, the data suggest that alcohol‐indued changes in glycosylation may contribute to increased risk of infections and cancer fatalities by compromising immune functions in individuals who misuse alcohol.

**Fig. 8:**
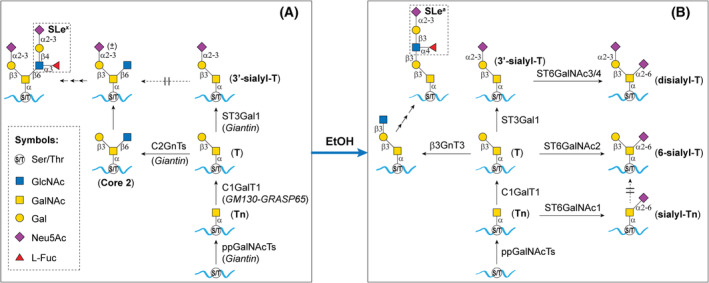
Altered biosynthesis of mucin O‐glycans in alcohol‐treated HTBE cells. (A) HTBE cells synthesize sLe^x^ extended from core 2 branch produced by C2GnT1. (B) In EtOH‐treated HTBE cells, loss of C2GnT1 coupled with shifted Golgi targeting of other glycosylation enzymes as the result of loss of giantin functions leads to loss of sLe^x^ but increase of Tn, sTn, T antigen, 6sT, DisT, and sLe^a^.

Mucin O‐glycans are glycans conjugated to Ser/Thr in glycoproteins via GalNAc. Biosynthesis of mucin O‐glycans occurs exclusively in the Golgi Apparatus. Figure 8A shows the biosynthesis pathway of sLe^x^ extended from core 2 branch in the Golgi of cells with functional giantin. Synthesis of mucin O‐glycans is initiated at the *cis*‐Golgi as catalyzed by peptidyl *N*‐acetylgalactosaminyltransferases (ppGalNAcTs) [47] to form Tn (GalNAcαSer/Thr). It is followed by the action of GalNAcαSer/Thr:β3galactosyltransferase 1 (C1GalT1) [48] to convert Tn to T antigen (Galβ3GalNAαSer/Thr). Then, the T antigen is extended by addition of GlcNAc to C6 of GalNAc to form core 2 structure [Galβ3(**GlcNAcβ6**)GalNAcαSer/Thr] as catalyzed by C2GnT1 [43]. The core 2 structure is further decorated with sLe^x^ by sequential additions of Gal, Neu5Ac, and Fuc as catalyzed by β4galactosyltransferases (β4GalTs) [49, 50], α2‐3sialyltransferases (ST3Gals) [50, 51], and α3fucosyltransferases (FuTs) [50, 52, 53], respectively. Some galactoses in the T antigens may be decorated with α2‐3Neu5Ac as catalyzed by α2‐3sialyltransferase 1 (ST3Gal1) [54, 55]. The glycosyltransferases (GTs) involved in the synthesis of mucin O‐glycans are synthesized in the ER and then transported by vesicles to the Golgi complexes [21]. Giantin [20, 21, 22, 25] and GM130‐GRASP65 [25, 56, 57, 58, 59, 60] are Golgi matrix proteins, which participate in the maintenance of Golgi morphology and serve as the docking sites for the ER‐derived vesicles that transport the glycosylation enzymes [21, 22, 23, 24, 25]. Giantin is the docking site for these vesicles except those that transport C1GalT1 in normal cells and benign cancer cells under basal conditions [21, 22, 23, 24, 25]. The GM130‐GRASP65 complex is the other Golgi docking site of the ER‐derived vesicles [21, 22, 25]. C1GalT1 is the only glycosylation enzyme which utilizes this site for Golgi targeting [21, 22, 25] while C2GnTs are the glycosylation enzymes which only utilize giantin for Golgi targeting [16, 17]. When cancer advances to the malignant stage, giantin is inactivated [21], which leads to loss of C2GnTs [22, 59] and shifts Golgi targeting of all other glycosylation enzymes to GM140‐GRASP65 site, culminating in the formation of tumor‐associated carbohydrate antigens (TACAs), which include Tn, sTn, T, sTs, and sLe^a^ as shown in Fig. 8B [29, 30, 31, 32, 33, 34].

We have shown that exposure of HTBE cells to 30 mm alcohol prevented C2GnT1 from reaching Golgi complexes without affecting Golgi morphology while significant Golgi fragmentation was detected at high (80–100 mm) alcohol concentrations (Figs 1 and 2). Because the cytoplasmic tail (MLRTLLRRRL) of C2GnT1 contains one of the dual basic amino acid sequences RR/RK/KK present in the cytoplasmic domain of the glycosylation enzymes that target the giantin site [25], one possible explanation of the failure of C2GnT1 to target the Golgi is the result of the modification of the arginine residues by ACH. This remains to be validated. However, in malignant cancer [22, 25] and alcohol‐treated hepatocytes [35, 36], Golgi fragmentation is the result of failure of giantin to form dimers.

Lack of the unique amino acid sequence in the cytoplasmic tail of C2GnT1 required for its docking at the GM130‐GRASP65 site prevents this enzyme from reaching the Golgi while other glycosylation enzymes can still reach the Golgi because their cytoplasmic tails also contain the unique amino acid sequences that help guide these enzymes to the GM130‐GRASP65 site [25]. Shifted Golgi targeting of these glycosylation enzymes to the GM130‐GRASP65 site, which competes against C1GalT1 for docking, down regulates the Golgi targeting efficiency of C1GalT1 [22]. Under the dysregulated glycosylation environment at the GM130‐GRASP65 site, reduced C1GalT1 in the Golgi [60] combined with reduced glycosylation efficiency of C1GalT1 and ST6GalNAc 1 [61] to convert Tn to T antigen and Tn to sTn, respectively, leaves some Tn unprocessed. It should be mentioned that the sTn generated cannot be processed further [62]. However, some of the T antigens produced can be converted to 3'sialyl‐T (**Neu5Acα2‐3**Galβ3GalNAc) by ST3Gal1 [54, 55], 6sT [Galβ3(**Neu5Acα2‐6**)GalNAc] by ST6GalNAc 2 [63], and DisT [**Neu5Acα2‐3**Galβ3(**Neu5Acα2‐6**)GalNAc] by ST3Gal1 [54, 55] and ST6GalNAc 3/4 [64, 65, 66]. Formation of 6sT and DisT is the result of loss of C2GnT1 due to its inability to use GM130‐GRASP65 for targeting [21, 22]. Loss of C2GnT1 leaves C6 of GalNAc in the T antigen open for incorporation of Neu5Ac at this site as catalyzed by ST6GalNAc 3/4 [64, 65, 66]. This effect also leads to loss of sLe^x^ because this glycotope can no longer be extended from core 2 branch [67]. Further, loss of giantin‐mediated Golgi targeting also results in the formation of sLe^a^, which requires Galβ3GalNAcαSer/Thr: β3N‐acetylglucosaminyltransferase 3 (β3GnT3) [68] to extend core 1 structure. However, it is rather intriguing that this glycotope is formed only after inactivation of giantin‐mediated Golgi targeting of glycosylation enzymes while β3GnT3 level is not affected by alcohol (Fig. S1). Because T antigen is the common substrate for C2GnT1 [43], β3GnT3 [68], and ST3Gal1 [54, 55], one possible explanation lies in the relative Golgi localization of these three GTs. Given that C2GnT1 is localized at *cis‐medial* Golgi and ST3Gal1 is localized at *medial*‐*trans* Golgi [54, 55], failure of β3GnT3 to extend core 1 structure in cells with functional giantin is likely due to its inability to encounter its T antigen substrate because this GT is localized behind ST3Gal1 in the Golgi. However, in cells without functioning giantin‐mediated Golgi targeting process, the dysregulated environment at the GM130‐GRASP65 site created by alcohol exposure renders β3GnT3 the opportunity to access the T antigen substrate and extend the core 1 structure to enable the synthesis of sLe^a^. It should be mentioned that the levels of each member of the TACAs induced by alcohol are determined by the relative levels of the glycosylation enzymes which participate in their synthesis in a cell type‐specific manner.

We also have found that α2‐6sialo mucin O‐glycans induced by alcohol kill U937 macrophages, which is medicated by Siglec 7. Siglec 7 is a member of the inhibitory Siglec family present in many immune cells [17, 69, 70, 71, 72]. Binding of Siglec 7 to its sialo mucin O‐glycan ligands can suppress the cytotoxicity of NK cells [16] and induce apoptosis of immune cells [15]. Because immune cells that express Siglec 7 are more powerful functionally than the counterparts without Siglec 7 [70], killing of the immune cells by α2‐6sialo mucin O‐glycans induced by alcohol can help shield benign cancer cells from immune surveillance and enable them to survive and thrive in alcohol abusers until they advance to the malignant stage. It has been reported that the Siglec 7(+) natural killer cells are reduced in patients with cancer [71], obesity [72], or HIV [73]. Understanding the extent of the alcohol effect on overall cellular immunity is essential for developing strategy to alleviate the alcohol toxicity, which may also be applicable to other diseases associated with compromised immunity.

The Siglec 7 ligands include not only α2‐6sialo mucin O‐glycans but also polysialic acid [17], which are found in N‐linked glycans, mucin O‐glycans, and gangliosides [74, 75, 76]. Polysialic acids exhibit higher Siglec 7 binding affinity than those of α2‐6sialo mucin O‐glycans [17]. GD2 and GD3 gangliosides have been shown to be elevated in cancer [77]. Also, the questions of whether the 6′sT and DisT‐like structures, −(±Neu5Acα2‐3)Galβ4(**Neu5Acα2‐6**)GalNAcβ3‐, present in gangliosides GD_1α_, GT_1bα_, and GQ_1bα_ [76] can serve as Siglec 7 ligands and whether alcohol can induce formation of these sialo glycans and polysialic acids such as GD2 and GD3 [77] remains to be examined. Further, current study only shows the effect of alcohol on the formation of α2‐6sialo mucin O‐glycans on the plasma membrane and their effect on immune cells. The effect of these sialo mucin O‐glycans and polysialic acids associated with secreted and shed glycoconjugates in circulation on the immune cells also awaits examination.

Finally, it should be mentioned that the alcohol toxic effects are medicated by its metabolite ACH, which can be prevented by inhibition of its formation or enhancement of its removal. The alcohol toxic effects also can be prevented by treatment with blebbistatin, an inhibitor of non‐muscle myosin IIA involved in Golgi fragmentation [22], suggesting that prevention of Golgi fragmentation can restore Golgi glycosylation function. Given that the α2‐6sialo mucin O‐glycans can suppress immune functions [15, 16, 17, 31, 32], they can help explain how alcohol abuse promotes cancer progression by protecting benign cancers from immune surveillance and enabling them to advance to malignant stage. Additionally, these sialo mucin O‐glycans induced by alcohol may contribute to bacterial infections by serving as the ligands for bacterial adhesins [78, 79, 80, 81]. The discovery made in this study can help guide future studies of the alcohol effects on cancer progression and bacterial infections, which may help develop strategies to prevent and/or treat illnesses caused by alcohol.

In conclusion, alcohol induces formation of α2‐6sialo mucin O‐glycans by a mechanism similar to the one employed by malignant cancer to generate same sialo mucin O‐glycans. The α2‐6sialo mucin O‐glycans induced by alcohol can kill immune cells which express Siglec 7. This alcohol effect can compromise immune function and lead to increased infections and cancer fatalities.

## Conflict of interest

The authors declare no conflict of interest.

### Peer review

The peer review history for this article is available at https://www.webofscience.com/api/gateway/wos/peer‐review/10.1002/2211‐5463.13919.

## Author contributions

PWC conceives the idea, supervises the experimental design and execution of the experiments, performs killing of U937 cells by alcohol‐treated HTE cells, interprets the results, and writes the manuscript. KB manages the preparation of the ALI cultures of HTBE cells. LL synthesizes sTn‐Ser and disialyl‐T‐Ser and prepares Fig. 8. DRS manages the cytotoxicity assay of U937 cells and performs statistical analysis and graphing. All authors participate in the editing of the manuscript and approve the final version of the article.

## Supporting information


**Fig. S1.** Alcohol does not affect β3GnT3 protein level.
**Table S1.** Alcohol increases Tn, T, sTn and sTs in human tracheobronchial cells.

## Data Availability

The datasets underlying this article will be shared upon a reasonable request to the corresponding author.
