# Supplementary material for: Alcohol induces α2‐6sialo mucin O‐glycans that kill U937 macrophages mediated by sialic acid‐binding immunoglobulin‐like lectin 7 (Siglec 7)
Source: FEBS Open Bio. 2024 Nov 26;15(1):165–79. doi: 10.1002/2211-5463.13919 (PMC11705458; doi:10.1002/2211-5463.13919)
Supplement: Supplementary file 1 — Fig. S1. Alcohol does not affect β3GnT3 protein level. Table S1. Alcohol increases Tn, T, sTn and sTs in human tracheobronchial cells. [file FEB4-15-165-s001.docx]

 Supplemental Table and Figure
